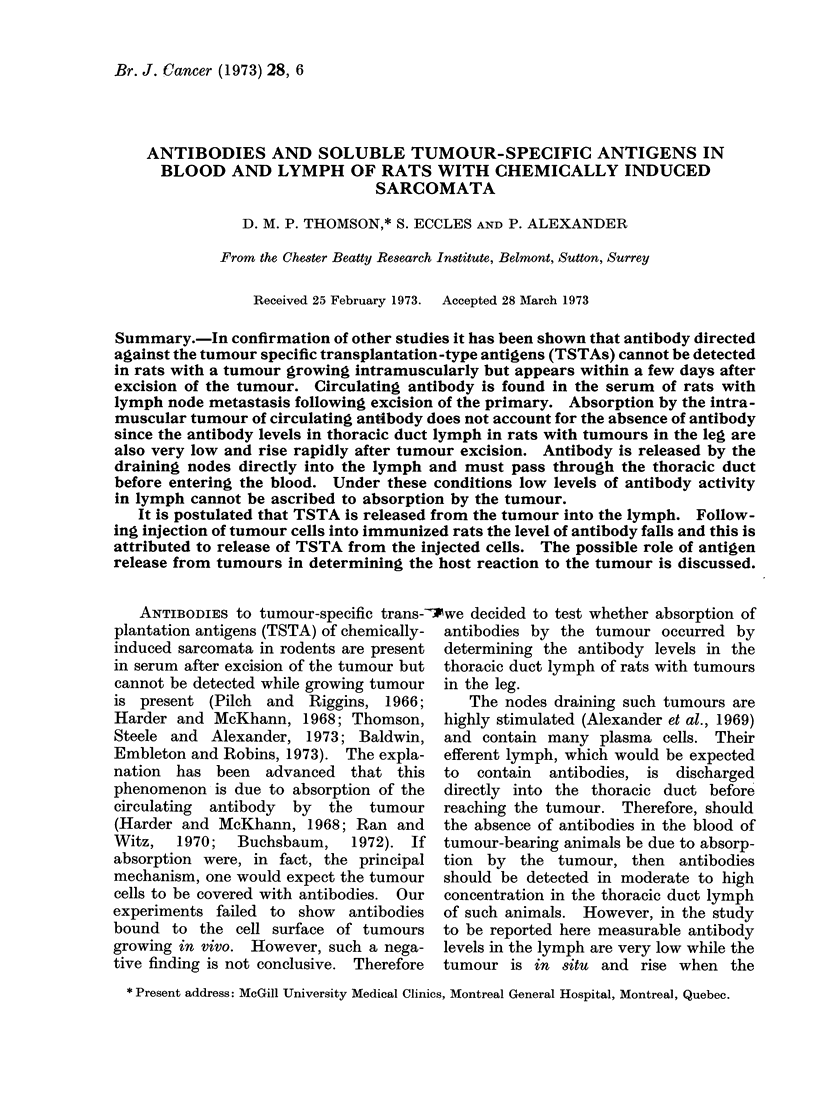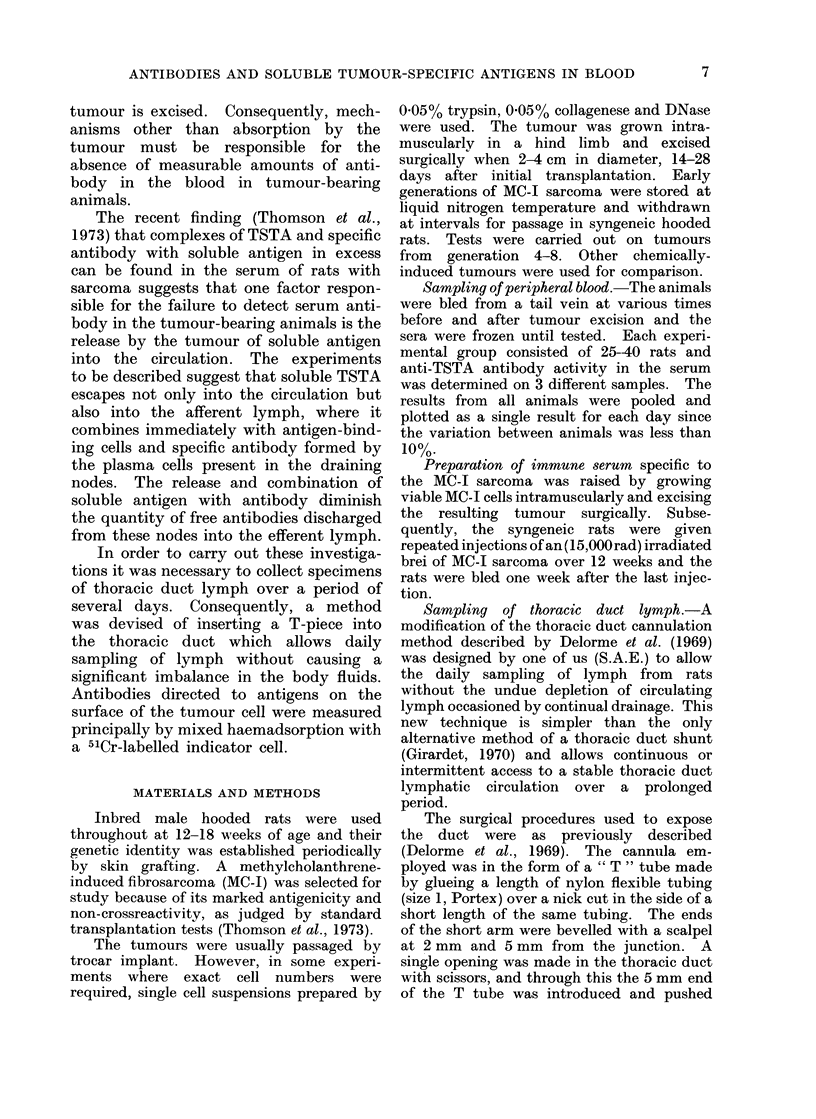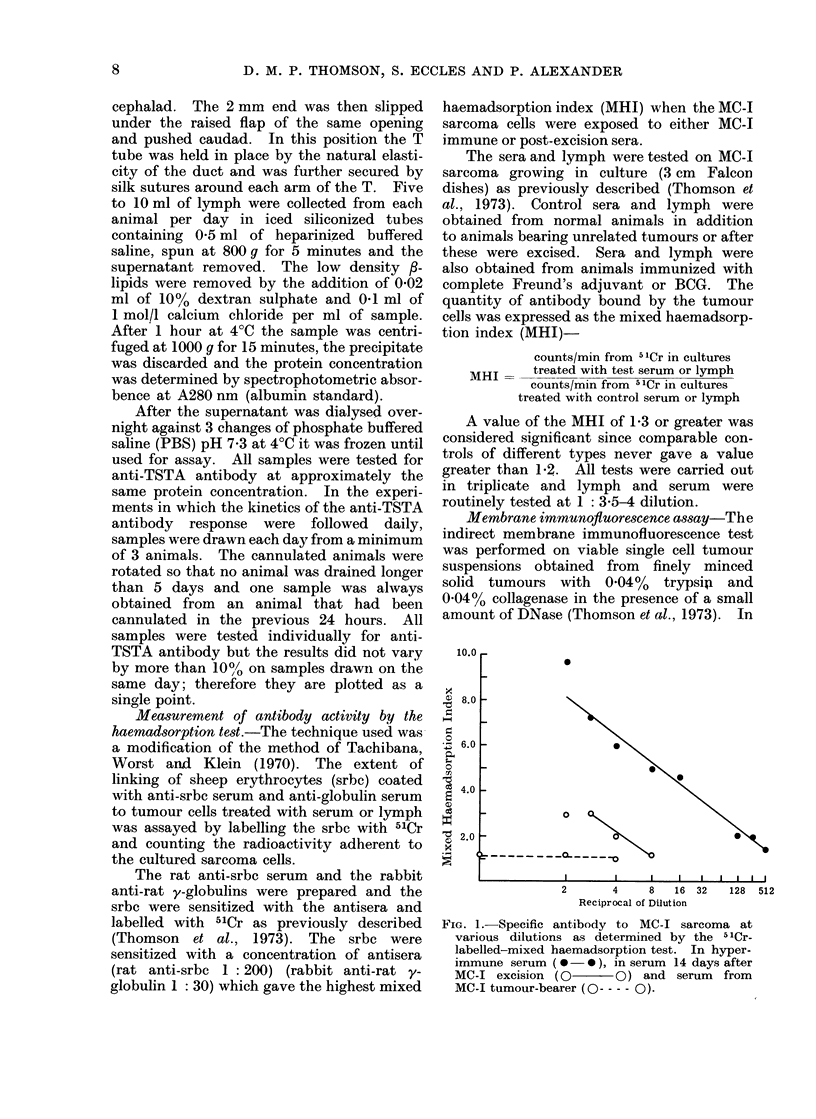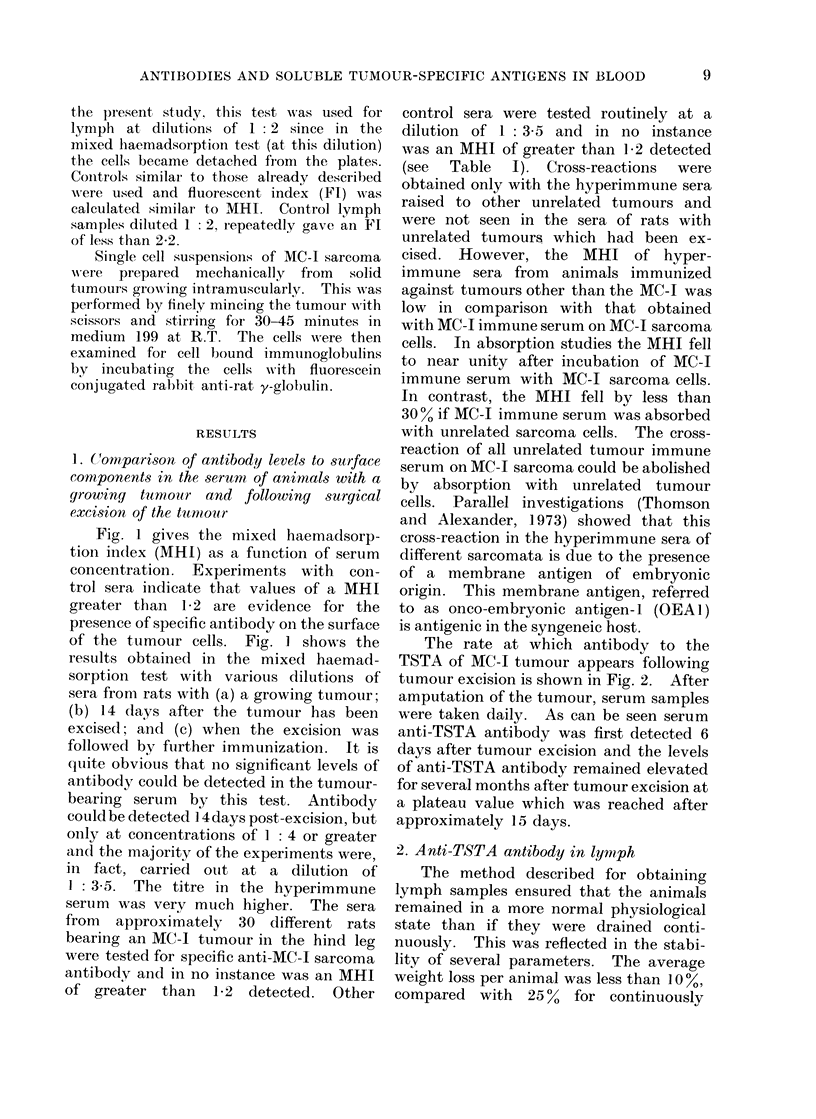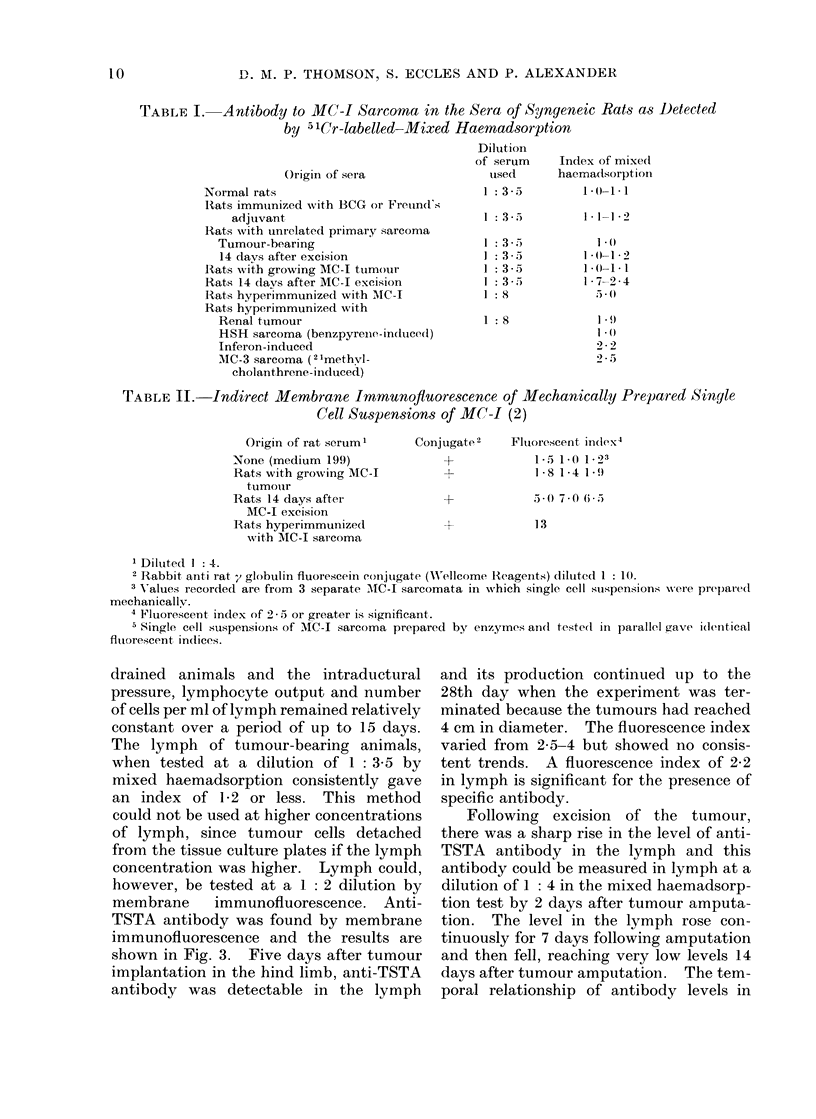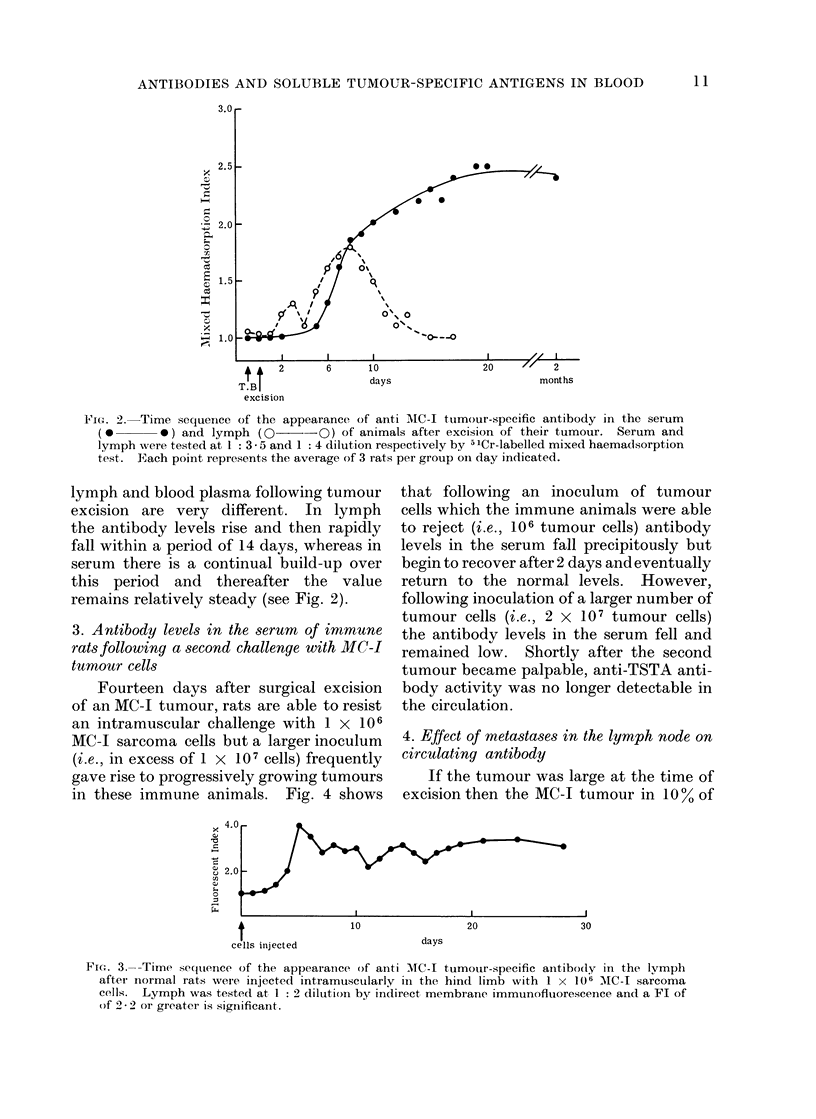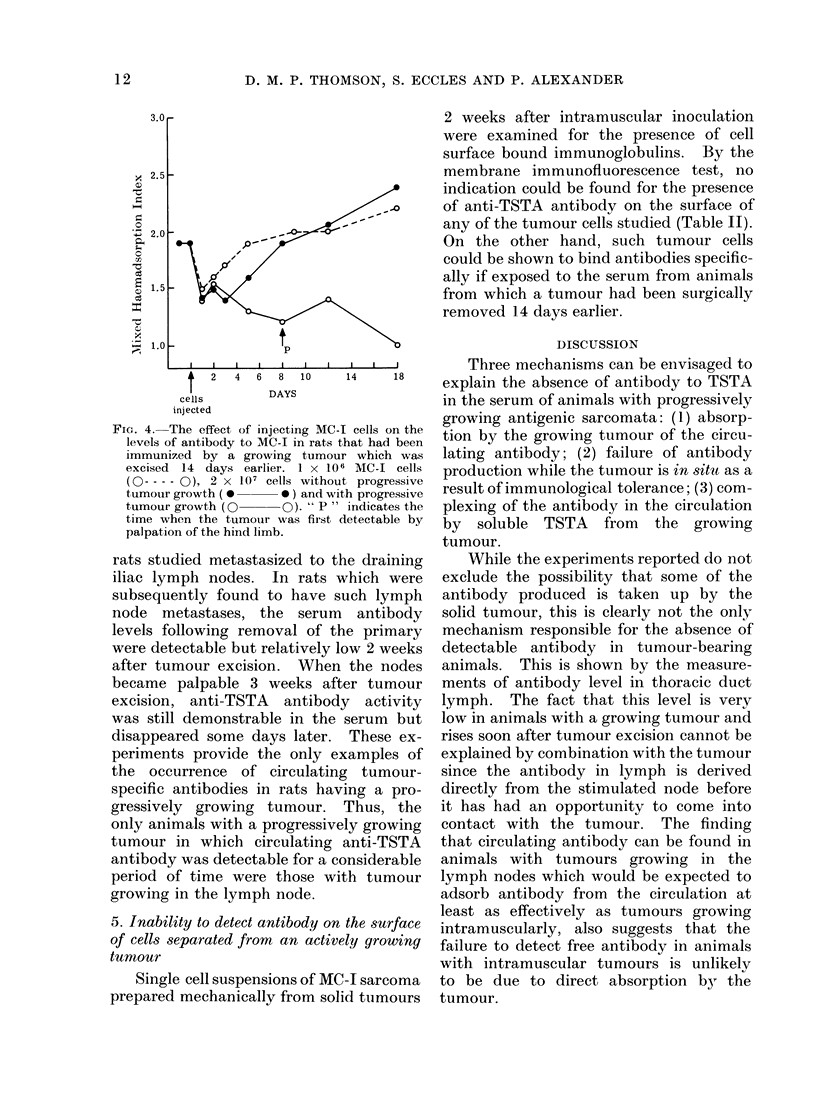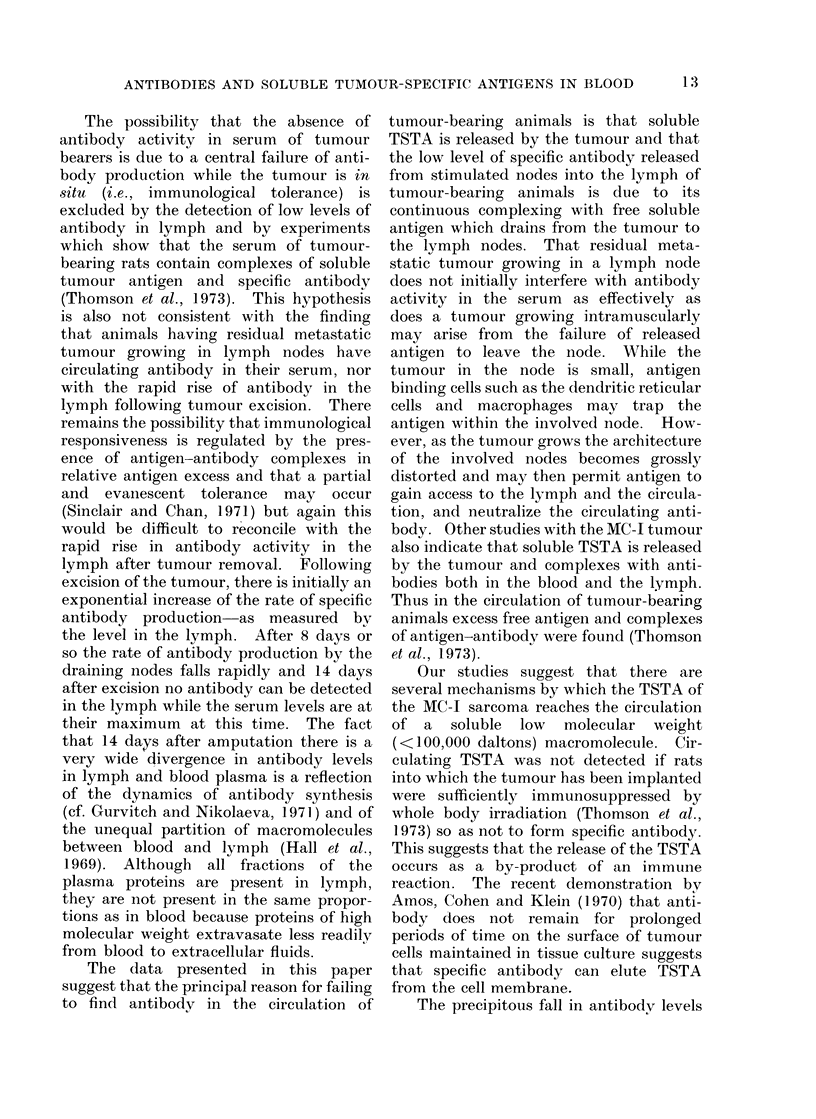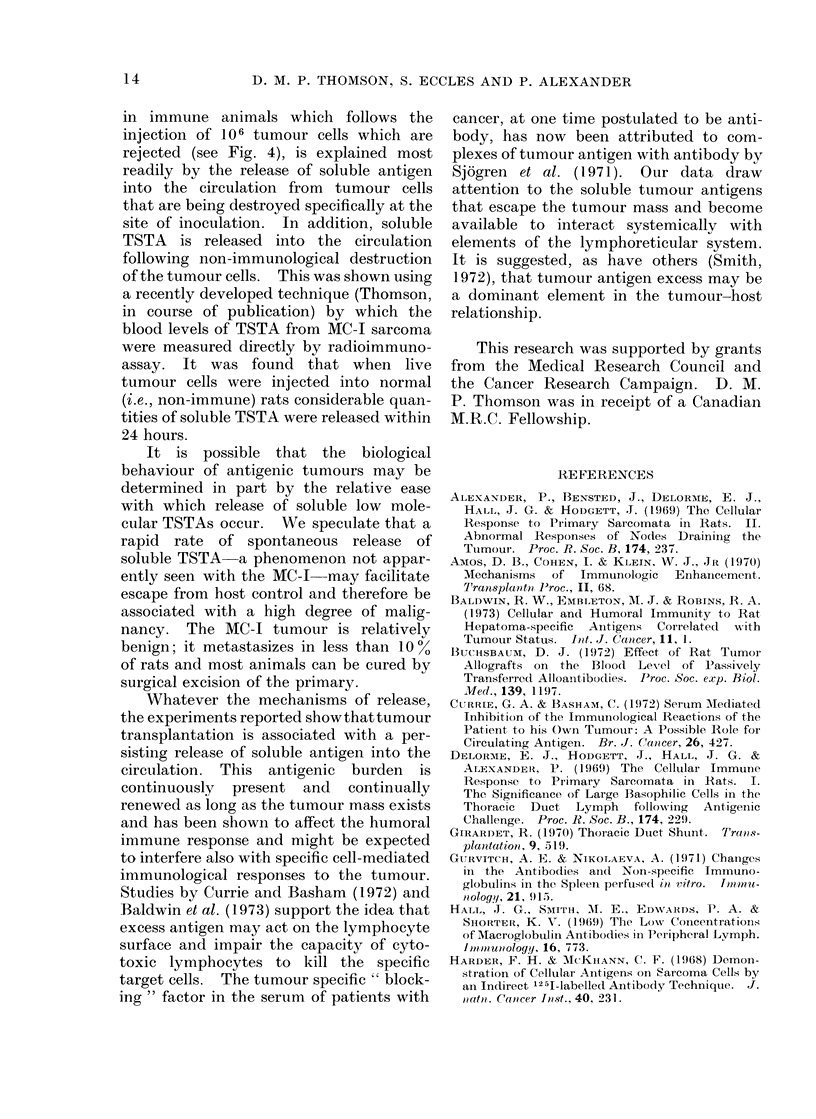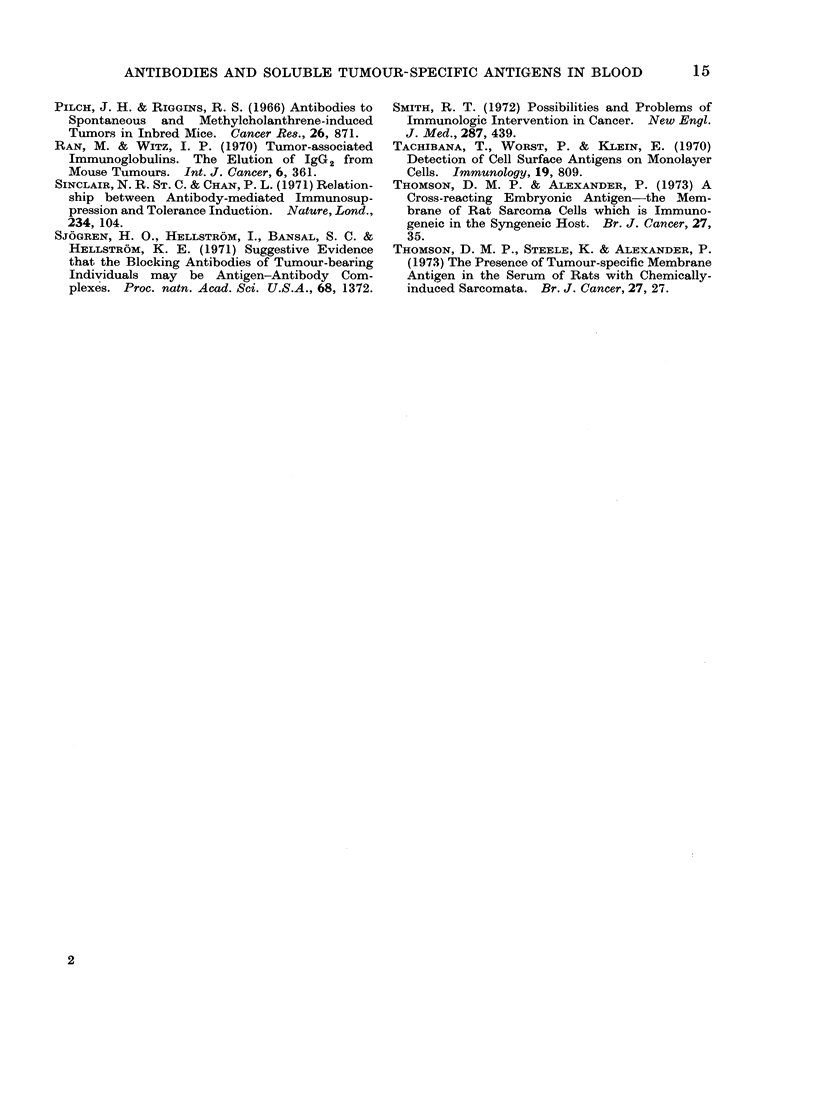# Antibodies and Soluble Tumour-specific Antigens in Blood and Lymph of Rats with Chemically Induced Sarcomata

**DOI:** 10.1038/bjc.1973.65

**Published:** 1973-07

**Authors:** D. M. P. Thomson, S. Eccles, P. Alexander

## Abstract

In confirmation of other studies it has been shown that antibody directed against the tumour specific transplantation-type antigens (TSTAs) cannot be detected in rats with a tumour growing intramuscularly but appears within a few days after excision of the tumour. Circulating antibody is found in the serum of rats with lymph node metastasis following excision of the primary. Absorption by the intramuscular tumour of circulating antibody does not account for the absence of antibody since the antibody levels in thoracic duct lymph in rats with tumours in the leg are also very low and rise rapidly after tumour excision. Antibody is released by the draining nodes directly into the lymph and must pass through the thoracic duct before entering the blood. Under these conditions low levels of antibody activity in lymph cannot be ascribed to absorption by the tumour.

It is postulated that TSTA is released from the tumour into the lymph. Following injection of tumour cells into immunized rats the level of antibody falls and this is attributed to release of TSTA from the injected cells. The possible role of antigen release from tumours in determining the host reaction to the tumour is discussed.


*Br. J. Cancer* (1973) **28**, 6

### ANTIBODIES AND SOLUBLE TUMOUR-SPECIFIC ANTIGENS IN BLOOD AND LYMPH OF RATS WITH CHEMICALLY INDUCED SARCOMATA

D. M. P. THOMSON,* S. ECCLES AND P. ALEXANDER

*From the Chester Beatty Research Institute, Belmont, Sutton, Surrey*

Received 25 February 1973. Accepted 28 March 1973

**Summary.—In confirmation of other studies it has been shown that antibody directed against the tumour specific transplantation-type antigens (TSTAs) cannot be detected in rats with a tumour growing intramuscularly but appears within a few days after excision of the tumour. Circulating antibody is found in the serum of rats with lymph node metastasis following excision of the primary. Absorption by the intramuscular tumour of circulating antibody does not account for the absence of antibody since the antibody levels in thoracic duct lymph in rats with tumours in the leg are also very low and rise rapidly after tumour excision. Antibody is released by the draining nodes directly into the lymph and must pass through the thoracic duct before entering the blood. Under these conditions low levels of antibody activity in lymph cannot be ascribed to absorption by the tumour.**

**It is postulated that TSTA is released from the tumour into the lymph. Following injection of tumour cells into immunized rats the level of antibody falls and this is attributed to release of TSTA from the injected cells. The possible role of antigen release from tumours in determining the host reaction to the tumour is discussed.**

ANTIBODIES to tumour-specific transplantation antigens (TSTA) of chemically-induced sarcomata in rodents are present in serum after excision of the tumour but cannot be detected while growing tumour is present (Pilch and Riggins, 1966; Harder and McKhann, 1968; Thomson, Steele and Alexander, 1973; Baldwin, Embleton and Robins, 1973). The explanation has been advanced that this phenomenon is due to absorption of the circulating antibody by the tumour (Harder and McKhann, 1968; Ran and Witz, 1970; Buchsbaum, 1972). If absorption were, in fact, the principal mechanism, one would expect the tumour cells to be covered with antibodies. Our experiments failed to show antibodies bound to the cell surface of tumours growing *in vivo*. However, such a negative finding is not conclusive. Therefore we decided to test whether absorption of antibodies by the tumour occurred by determining the antibody levels in the thoracic duct lymph of rats with tumours in the leg.

The nodes draining such tumours are highly stimulated (Alexander *et al.*, 1969) and contain many plasma cells. Their efferent lymph, which would be expected to contain antibodies, is discharged directly into the thoracic duct before reaching the tumour. Therefore, should the absence of antibodies in the blood of tumour-bearing animals be due to absorption by the tumour, then antibodies should be detected in moderate to high concentration in the thoracic duct lymph of such animals. However, in the study to be reported here measurable antibody levels in the lymph are very low while the tumour is *in situ* and rise when the

* Present address: McGill University Medical Clinics, Montreal General Hospital, Montreal, Quebec.

tumour is excised. Consequently, mechanisms other than absorption by the tumour must be responsible for the absence of measurable amounts of antibody in the blood in tumour-bearing animals.

The recent finding (Thomson *et al.*, 1973) that complexes of TSTA and specific antibody with soluble antigen in excess can be found in the serum of rats with sarcoma suggests that one factor responsible for the failure to detect serum antibody in the tumour-bearing animals is the release by the tumour of soluble antigen into the circulation. The experiments to be described suggest that soluble TSTA escapes not only into the circulation but also into the afferent lymph, where it combines immediately with antigen-binding cells and specific antibody formed by the plasma cells present in the draining nodes. The release and combination of soluble antigen with antibody diminish the quantity of free antibodies discharged from these nodes into the efferent lymph.

In order to carry out these investigations it was necessary to collect specimens of thoracic duct lymph over a period of several days. Consequently, a method was devised of inserting a T-piece into the thoracic duct which allows daily sampling of lymph without causing a significant imbalance in the body fluids. Antibodies directed to antigens on the surface of the tumour cell were measured principally by mixed haemadsorption with a $^{51}$Cr-labelled indicator cell.

### MATERIALS AND METHODS

Inbred male hooded rats were used throughout at 12–18 weeks of age and their genetic identity was established periodically by skin grafting. A methylcholanthrene-induced fibrosarcoma (MC-I) was selected for study because of its marked antigenicity and non-crossreactivity, as judged by standard transplantation tests (Thomson *et al.*, 1973).

The tumours were usually passaged by trocar implant. However, in some experiments where exact cell numbers were required, single cell suspensions prepared by 0·05% trypsin, 0·05% collagenese and DNase were used. The tumour was grown intramuscularly in a hind limb and excised surgically when 2–4 cm in diameter, 14–28 days after initial transplantation. Early generations of MC-I sarcoma were stored at liquid nitrogen temperature and withdrawn at intervals for passage in syngeneic hooded rats. Tests were carried out on tumours from generation 4–8. Other chemically-induced tumours were used for comparison.

*Sampling of peripheral blood.*—The animals were bled from a tail vein at various times before and after tumour excision and the sera were frozen until tested. Each experimental group consisted of 25–40 rats and anti-TSTA antibody activity in the serum was determined on 3 different samples. The results from all animals were pooled and plotted as a single result for each day since the variation between animals was less than 10%.

*Preparation of immune serum* specific to the MC-I sarcoma was raised by growing viable MC-I cells intramuscularly and excising the resulting tumour surgically. Subsequently, the syngeneic rats were given repeated injections of an (15,000 rad) irradiated brei of MC-I sarcoma over 12 weeks and the rats were bled one week after the last injection.

*Sampling of thoracic duct lymph.*—A modification of the thoracic duct cannulation method described by Delorme *et al.* (1969) was designed by one of us (S.A.E.) to allow the daily sampling of lymph from rats without the undue depletion of circulating lymph occasioned by continual drainage. This new technique is simpler than the only alternative method of a thoracic duct shunt (Girardet, 1970) and allows continuous or intermittent access to a stable thoracic duct lymphatic circulation over a prolonged period.

The surgical procedures used to expose the duct were as previously described (Delorme *et al.*, 1969). The cannula employed was in the form of a " T " tube made by glueing a length of nylon flexible tubing (size 1, Portex) over a nick cut in the side of a short length of the same tubing. The ends of the short arm were bevelled with a scalpel at 2 mm and 5 mm from the junction. A single opening was made in the thoracic duct with scissors, and through this the 5 mm end of the T tube was introduced and pushed cephalad. The 2 mm end was then slipped under the raised flap of the same opening and pushed caudad. In this position the T tube was held in place by the natural elasticity of the duct and was further secured by silk sutures around each arm of the T. Five to 10 ml of lymph were collected from each animal per day in iced siliconized tubes containing 0·5 ml of heparinized buffered saline, spun at 800 *g* for 5 minutes and the supernatant removed. The low density $\beta$-lipids were removed by the addition of 0·02 ml of 10% dextran sulphate and 0·1 ml of 1 mol/l calcium chloride per ml of sample. After 1 hour at 4°C the sample was centrifuged at 1000 *g* for 15 minutes, the precipitate was discarded and the protein concentration was determined by spectrophotometric absorbence at A280 nm (albumin standard).

After the supernatant was dialysed overnight against 3 changes of phosphate buffered saline (PBS) pH 7·3 at 4°C it was frozen until used for assay. All samples were tested for anti-TSTA antibody at approximately the same protein concentration. In the experiments in which the kinetics of the anti-TSTA antibody response were followed daily, samples were drawn each day from a minimum of 3 animals. The cannulated animals were rotated so that no animal was drained longer than 5 days and one sample was always obtained from an animal that had been cannulated in the previous 24 hours. All samples were tested individually for anti-TSTA antibody but the results did not vary by more than 10% on samples drawn on the same day; therefore they are plotted as a single point.

*Measurement of antibody activity by the haemadsorption test.*—The technique used was a modification of the method of Tachibana, Worst and Klein (1970). The extent of linking of sheep erythrocytes (srbc) coated with anti-srbc serum and anti-globulin serum to tumour cells treated with serum or lymph was assayed by labelling the srbc with $^{51}Cr$ and counting the radioactivity adherent to the cultured sarcoma cells.

The rat anti-srbc serum and the rabbit anti-rat $\gamma$-globulins were prepared and the srbc were sensitized with the antisera and labelled with $^{51}Cr$ as previously described (Thomson *et al.*, 1973). The srbc were sensitized with a concentration of antisera (rat anti-srbc 1 : 200) (rabbit anti-rat $\gamma$-globulin 1 : 30) which gave the highest mixed haemadsorption index (MHI) when the MC-I sarcoma cells were exposed to either MC-I immune or post-excision sera.

The sera and lymph were tested on MC-I sarcoma growing in culture (3 cm Falcon dishes) as previously described (Thomson *et al.*, 1973). Control sera and lymph were obtained from normal animals in addition to animals bearing unrelated tumours or after these were excised. Sera and lymph were also obtained from animals immunized with complete Freund's adjuvant or BCG. The quantity of antibody bound by the tumour cells was expressed as the mixed haemadsorption index (MHI)—

$$\text{MHI} = \frac{\text{counts/min from } ^{51}\text{Cr in cultures treated with test serum or lymph}}{\text{counts/min from } ^{51}\text{Cr in cultures treated with control serum or lymph}}$$

A value of the MHI of 1·3 or greater was considered significant since comparable controls of different types never gave a value greater than 1·2. All tests were carried out in triplicate and lymph and serum were routinely tested at 1 : 3·5–4 dilution.

*Membrane immunofluorescence assay*—The indirect membrane immunofluorescence test was performed on viable single cell tumour suspensions obtained from finely minced solid tumours with 0·04% trypsin and 0·04% collagenase in the presence of a small amount of DNase (Thomson *et al.*, 1973). In

FIG. 1.—Specific antibody to MC-I sarcoma at various dilutions as determined by the $^{51}$Cr-labelled–mixed haemadsorption test. In hyperimmune serum (●—●), in serum 14 days after MC-I excision (○——○) and serum from MC-I tumour-bearer (○- - - ○).

the present study, this test was used for lymph at dilutions of 1 : 2 since in the mixed haemadsorption test (at this dilution) the cells became detached from the plates. Controls similar to those already described were used and fluorescent index (FI) was calculated similar to MHI. Control lymph samples diluted 1 : 2, repeatedly gave an FI of less than 2·2.

Single cell suspensions of MC-I sarcoma were prepared mechanically from solid tumours growing intramuscularly. This was performed by finely mincing the tumour with scissors and stirring for 30–45 minutes in medium 199 at R.T. The cells were then examined for cell bound immunoglobulins by incubating the cells with fluorescein conjugated rabbit anti-rat $\gamma$-globulin.

### RESULTS

### 1. *Comparison of antibody levels to surface components in the serum of animals with a growing tumour and following surgical excision of the tumour*

Fig. 1 gives the mixed haemadsorption index (MHI) as a function of serum concentration. Experiments with control sera indicate that values of a MHI greater than 1·2 are evidence for the presence of specific antibody on the surface of the tumour cells. Fig. 1 shows the results obtained in the mixed haemadsorption test with various dilutions of sera from rats with (a) a growing tumour; (b) 14 days after the tumour has been excised; and (c) when the excision was followed by further immunization. It is quite obvious that no significant levels of antibody could be detected in the tumour-bearing serum by this test. Antibody could be detected 14 days post-excision, but only at concentrations of 1 : 4 or greater and the majority of the experiments were, in fact, carried out at a dilution of 1 : 3·5. The titre in the hyperimmune serum was very much higher. The sera from approximately 30 different rats bearing an MC-I tumour in the hind leg were tested for specific anti-MC-I sarcoma antibody and in no instance was an MHI of greater than 1·2 detected. Other control sera were tested routinely at a dilution of 1 : 3·5 and in no instance was an MHI of greater than 1·2 detected (see Table I). Cross-reactions were obtained only with the hyperimmune sera raised to other unrelated tumours and were not seen in the sera of rats with unrelated tumours which had been excised. However, the MHI of hyperimmune sera from animals immunized against tumours other than the MC-I was low in comparison with that obtained with MC-I immune serum on MC-I sarcoma cells. In absorption studies the MHI fell to near unity after incubation of MC-I immune serum with MC-I sarcoma cells. In contrast, the MHI fell by less than 30% if MC-I immune serum was absorbed with unrelated sarcoma cells. The cross-reaction of all unrelated tumour immune serum on MC-I sarcoma could be abolished by absorption with unrelated tumour cells. Parallel investigations (Thomson and Alexander, 1973) showed that this cross-reaction in the hyperimmune sera of different sarcomata is due to the presence of a membrane antigen of embryonic origin. This membrane antigen, referred to as onco-embryonic antigen-1 (OEA1) is antigenic in the syngeneic host.

The rate at which antibody to the TSTA of MC-I tumour appears following tumour excision is shown in Fig. 2. After amputation of the tumour, serum samples were taken daily. As can be seen serum anti-TSTA antibody was first detected 6 days after tumour excision and the levels of anti-TSTA antibody remained elevated for several months after tumour excision at a plateau value which was reached after approximately 15 days.

### 2. *Anti-TSTA antibody in lymph*

The method described for obtaining lymph samples ensured that the animals remained in a more normal physiological state than if they were drained continuously. This was reflected in the stability of several parameters. The average weight loss per animal was less than 10%, compared with 25% for continuously

TABLE I.—*Antibody to MC-I Sarcoma in the Sera of Syngeneic Rats as Detected by $^{51}Cr$-labelled–Mixed Haemadsorption*

| Origin of sera | Dilution of serum used | Index of mixed haemadsorption |
|---|---|---|
| Normal rats | 1 : 3·5 | 1·0–1·1 |
| Rats immunized with BCG or Freund's adjuvant | 1 : 3·5 | 1·1–1·2 |
| Rats with unrelated primary sarcoma | | |
| Tumour-bearing | 1 : 3·5 | 1·0 |
| 14 days after excision | 1 : 3·5 | 1·0–1·2 |
| Rats with growing MC-I tumour | 1 : 3·5 | 1·0–1·1 |
| Rats 14 days after MC-I excision | 1 : 3·5 | 1·7–2·4 |
| Rats hyperimmunized with MC-I | 1 : 8 | 5·0 |
| Rats hyperimmunized with | | |
| Renal tumour | 1 : 8 | 1·9 |
| HSH sarcoma (benzpyrene-induced) | | 1·0 |
| Inferon-induced | | 2·2 |
| MC-3 sarcoma ($^{21}$methyl-cholanthrene-induced) | | 2·5 |

TABLE II.—*Indirect Membrane Immunofluorescence of Mechanically Prepared Single Cell Suspensions of MC-I* (2)

| Origin of rat serum[1] | Conjugate[2] | Fluorescent index[4] |
|---|---|---|
| None (medium 199) | + | 1·5 1·0 1·2[3] |
| Rats with growing MC-I tumour | + | 1·8 1·4 1·9 |
| Rats 14 days after MC-I excision | + | 5·0 7·0 6·5 |
| Rats hyperimmunized with MC-I sarcoma | + | 13 |

[1] Diluted 1 : 4.

[2] Rabbit anti rat $\gamma$ globulin fluorescein conjugate (Wellcome Reagents) diluted 1 : 10.

[3] Values recorded are from 3 separate MC-I sarcomata in which single cell suspensions were prepared mechanically.

[4] Fluorescent index of 2·5 or greater is significant.

[5] Single cell suspensions of MC-I sarcoma prepared by enzymes and tested in parallel gave identical fluorescent indices.

drained animals and the intraductural pressure, lymphocyte output and number of cells per ml of lymph remained relatively constant over a period of up to 15 days. The lymph of tumour-bearing animals, when tested at a dilution of 1 : 3·5 by mixed haemadsorption consistently gave an index of 1·2 or less. This method could not be used at higher concentrations of lymph, since tumour cells detached from the tissue culture plates if the lymph concentration was higher. Lymph could, however, be tested at a 1 : 2 dilution by membrane immunofluorescence. Anti-TSTA antibody was found by membrane immunofluorescence and the results are shown in Fig. 3. Five days after tumour implantation in the hind limb, anti-TSTA antibody was detectable in the lymph and its production continued up to the 28th day when the experiment was terminated because the tumours had reached 4 cm in diameter. The fluorescence index varied from 2·5–4 but showed no consistent trends. A fluorescence index of 2·2 in lymph is significant for the presence of specific antibody.

Following excision of the tumour, there was a sharp rise in the level of anti-TSTA antibody in the lymph and this antibody could be measured in lymph at a dilution of 1 : 4 in the mixed haemadsorption test by 2 days after tumour amputation. The level in the lymph rose continuously for 7 days following amputation and then fell, reaching very low levels 14 days after tumour amputation. The temporal relationship of antibody levels in

FIG. 2.—Time sequence of the appearance of anti MC-I tumour-specific antibody in the serum (● ——— ●) and lymph (○ ——— ○) of animals after excision of their tumour. Serum and lymph were tested at 1 : 3·5 and 1 : 4 dilution respectively by $^{51}$Cr-labelled mixed haemadsorption test. Each point represents the average of 3 rats per group on day indicated.

lymph and blood plasma following tumour excision are very different. In lymph the antibody levels rise and then rapidly fall within a period of 14 days, whereas in serum there is a continual build-up over this period and thereafter the value remains relatively steady (see Fig. 2).

3. *Antibody levels in the serum of immune rats following a second challenge with MC-I tumour cells*

Fourteen days after surgical excision of an MC-I tumour, rats are able to resist an intramuscular challenge with $1 \times 10^6$ MC-I sarcoma cells but a larger inoculum (*i.e.*, in excess of $1 \times 10^7$ cells) frequently gave rise to progressively growing tumours in these immune animals. Fig. 4 shows that following an inoculum of tumour cells which the immune animals were able to reject (*i.e.*, $10^6$ tumour cells) antibody levels in the serum fall precipitously but begin to recover after 2 days and eventually return to the normal levels. However, following inoculation of a larger number of tumour cells (*i.e.*, $2 \times 10^7$ tumour cells) the antibody levels in the serum fell and remained low. Shortly after the second tumour became palpable, anti-TSTA antibody activity was no longer detectable in the circulation.

4. *Effect of metastases in the lymph node on circulating antibody*

If the tumour was large at the time of excision then the MC-I tumour in 10% of

FIG. 3.—Time sequence of the appearance of anti MC-I tumour-specific antibody in the lymph after normal rats were injected intramuscularly in the hind limb with $1 \times 10^6$ MC-I sarcoma cells. Lymph was tested at 1 : 2 dilution by indirect membrane immunofluorescence and a FI of of 2·2 or greater is significant.

FIG. 4.—The effect of injecting MC-I cells on the levels of antibody to MC-I in rats that had been immunized by a growing tumour which was excised 14 days earlier. $1 \times 10^6$ MC-I cells (○- - - - ○), $2 \times 10^7$ cells without progressive tumour growth (●———●) and with progressive tumour growth (○———○). " P " indicates the time when the tumour was first detectable by palpation of the hind limb.

rats studied metastasized to the draining iliac lymph nodes. In rats which were subsequently found to have such lymph node metastases, the serum antibody levels following removal of the primary were detectable but relatively low 2 weeks after tumour excision. When the nodes became palpable 3 weeks after tumour excision, anti-TSTA antibody activity was still demonstrable in the serum but disappeared some days later. These experiments provide the only examples of the occurrence of circulating tumour-specific antibodies in rats having a progressively growing tumour. Thus, the only animals with a progressively growing tumour in which circulating anti-TSTA antibody was detectable for a considerable period of time were those with tumour growing in the lymph node.

### 5. *Inability to detect antibody on the surface of cells separated from an actively growing tumour*

Single cell suspensions of MC-I sarcoma prepared mechanically from solid tumours 2 weeks after intramuscular inoculation were examined for the presence of cell surface bound immunoglobulins. By the membrane immunofluorescence test, no indication could be found for the presence of anti-TSTA antibody on the surface of any of the tumour cells studied (Table II). On the other hand, such tumour cells could be shown to bind antibodies specifically if exposed to the serum from animals from which a tumour had been surgically removed 14 days earlier.

### DISCUSSION

Three mechanisms can be envisaged to explain the absence of antibody to TSTA in the serum of animals with progressively growing antigenic sarcomata: (1) absorption by the growing tumour of the circulating antibody; (2) failure of antibody production while the tumour is *in situ* as a result of immunological tolerance; (3) complexing of the antibody in the circulation by soluble TSTA from the growing tumour.

While the experiments reported do not exclude the possibility that some of the antibody produced is taken up by the solid tumour, this is clearly not the only mechanism responsible for the absence of detectable antibody in tumour-bearing animals. This is shown by the measurements of antibody level in thoracic duct lymph. The fact that this level is very low in animals with a growing tumour and rises soon after tumour excision cannot be explained by combination with the tumour since the antibody in lymph is derived directly from the stimulated node before it has had an opportunity to come into contact with the tumour. The finding that circulating antibody can be found in animals with tumours growing in the lymph nodes which would be expected to adsorb antibody from the circulation at least as effectively as tumours growing intramuscularly, also suggests that the failure to detect free antibody in animals with intramuscular tumours is unlikely to be due to direct absorption by the tumour.

The possibility that the absence of antibody activity in serum of tumour bearers is due to a central failure of antibody production while the tumour is *in situ* (*i.e.*, immunological tolerance) is excluded by the detection of low levels of antibody in lymph and by experiments which show that the serum of tumour-bearing rats contain complexes of soluble tumour antigen and specific antibody (Thomson *et al.*, 1973). This hypothesis is also not consistent with the finding that animals having residual metastatic tumour growing in lymph nodes have circulating antibody in their serum, nor with the rapid rise of antibody in the lymph following tumour excision. There remains the possibility that immunological responsiveness is regulated by the presence of antigen–antibody complexes in relative antigen excess and that a partial and evanescent tolerance may occur (Sinclair and Chan, 1971) but again this would be difficult to reconcile with the rapid rise in antibody activity in the lymph after tumour removal. Following excision of the tumour, there is initially an exponential increase of the rate of specific antibody production—as measured by the level in the lymph. After 8 days or so the rate of antibody production by the draining nodes falls rapidly and 14 days after excision no antibody can be detected in the lymph while the serum levels are at their maximum at this time. The fact that 14 days after amputation there is a very wide divergence in antibody levels in lymph and blood plasma is a reflection of the dynamics of antibody synthesis (cf. Gurvitch and Nikolaeva, 1971) and of the unequal partition of macromolecules between blood and lymph (Hall *et al.*, 1969). Although all fractions of the plasma proteins are present in lymph, they are not present in the same proportions as in blood because proteins of high molecular weight extravasate less readily from blood to extracellular fluids.

The data presented in this paper suggest that the principal reason for failing to find antibody in the circulation of tumour-bearing animals is that soluble TSTA is released by the tumour and that the low level of specific antibody released from stimulated nodes into the lymph of tumour-bearing animals is due to its continuous complexing with free soluble antigen which drains from the tumour to the lymph nodes. That residual metastatic tumour growing in a lymph node does not initially interfere with antibody activity in the serum as effectively as does a tumour growing intramuscularly may arise from the failure of released antigen to leave the node. While the tumour in the node is small, antigen binding cells such as the dendritic reticular cells and macrophages may trap the antigen within the involved node. However, as the tumour grows the architecture of the involved nodes becomes grossly distorted and may then permit antigen to gain access to the lymph and the circulation, and neutralize the circulating antibody. Other studies with the MC-I tumour also indicate that soluble TSTA is released by the tumour and complexes with antibodies both in the blood and the lymph. Thus in the circulation of tumour-bearing animals excess free antigen and complexes of antigen–antibody were found (Thomson *et al.*, 1973).

Our studies suggest that there are several mechanisms by which the TSTA of the MC-I sarcoma reaches the circulation of a soluble low molecular weight (<100,000 daltons) macromolecule. Circulating TSTA was not detected if rats into which the tumour has been implanted were sufficiently immunosuppressed by whole body irradiation (Thomson *et al.*, 1973) so as not to form specific antibody. This suggests that the release of the TSTA occurs as a by-product of an immune reaction. The recent demonstration by Amos, Cohen and Klein (1970) that antibody does not remain for prolonged periods of time on the surface of tumour cells maintained in tissue culture suggests that specific antibody can elute TSTA from the cell membrane.

The precipitous fall in antibody levels in immune animals which follows the injection of $10^6$ tumour cells which are rejected (see Fig. 4), is explained most readily by the release of soluble antigen into the circulation from tumour cells that are being destroyed specifically at the site of inoculation. In addition, soluble TSTA is released into the circulation following non-immunological destruction of the tumour cells. This was shown using a recently developed technique (Thomson, in course of publication) by which the blood levels of TSTA from MC-I sarcoma were measured directly by radioimmunoassay. It was found that when live tumour cells were injected into normal (*i.e.*, non-immune) rats considerable quantities of soluble TSTA were released within 24 hours.

It is possible that the biological behaviour of antigenic tumours may be determined in part by the relative ease with which release of soluble low molecular TSTAs occur. We speculate that a rapid rate of spontaneous release of soluble TSTA—a phenomenon not apparently seen with the MC-I—may facilitate escape from host control and therefore be associated with a high degree of malignancy. The MC-I tumour is relatively benign; it metastasizes in less than 10% of rats and most animals can be cured by surgical excision of the primary.

Whatever the mechanisms of release, the experiments reported show that tumour transplantation is associated with a persisting release of soluble antigen into the circulation. This antigenic burden is continuously present and continually renewed as long as the tumour mass exists and has been shown to affect the humoral immune response and might be expected to interfere also with specific cell-mediated immunological responses to the tumour. Studies by Currie and Basham (1972) and Baldwin *et al.* (1973) support the idea that excess antigen may act on the lymphocyte surface and impair the capacity of cytotoxic lymphocytes to kill the specific target cells. The tumour specific " blocking " factor in the serum of patients with cancer, at one time postulated to be antibody, has now been attributed to complexes of tumour antigen with antibody by Sjögren *et al.* (1971). Our data draw attention to the soluble tumour antigens that escape the tumour mass and become available to interact systemically with elements of the lymphoreticular system. It is suggested, as have others (Smith, 1972), that tumour antigen excess may be a dominant element in the tumour–host relationship.

This research was supported by grants from the Medical Research Council and the Cancer Research Campaign. D. M. P. Thomson was in receipt of a Canadian M.R.C. Fellowship.

### REFERENCES

Alexander, P., Bensted, J., Delorme, E. J., Hall, J. G. & Hodgett, J. (1969) The Cellular Response to Primary Sarcomata in Rats. II. Abnormal Responses of Nodes Draining the Tumour. *Proc. R. Soc. B*, **174**, 237.

Amos, D. B., Cohen, I. & Klein, W. J., Jr (1970) Mechanisms of Immunologic Enhancement. *Transplantn Proc.*, **II**, 68.

Baldwin, R. W., Embleton, M. J. & Robins, R. A. (1973) Cellular and Humoral Immunity to Rat Hepatoma-specific Antigens Correlated with Tumour Status. *Int. J. Cancer*, **11**, 1.

Buchsbaum, D. J. (1972) Effect of Rat Tumor Allografts on the Blood Level of Passively Transferred Alloantibodies. *Proc. Soc. exp. Biol. Med.*, **139**, 1197.

Currie, G. A. & Basham, C. (1972) Serum Mediated Inhibition of the Immunological Reactions of the Patient to his Own Tumour: A Possible Role for Circulating Antigen. *Br. J. Cancer*, **26**, 427.

Delorme, E. J., Hodgett, J., Hall, J. G. & Alexander, P. (1969) The Cellular Immune Response to Primary Sarcomata in Rats. I. The Significance of Large Basophilic Cells in the Thoracic Duct Lymph following Antigenic Challenge. *Proc. R. Soc. B.*, **174**, 229.

Girardet, R. (1970) Thoracic Duct Shunt. *Transplantation*, **9**, 519.

Gurvitch, A. E. & Nikolaeva, A. (1971) Changes in the Antibodies and Non-specific Immunoglobulins in the Spleen perfused *in vitro*. *Immunology*, **21**, 915.

Hall, J. G., Smith, M. E., Edwards, P. A. & Shorter, K. V. (1969) The Low Concentrations of Macroglobulin Antibodies in Peripheral Lymph. *Immunology*, **16**, 773.

Harder, F. H. & McKhann, C. F. (1968) Demonstration of Cellular Antigens on Sarcoma Cells by an Indirect $^{125}$I-labelled Antibody Technique. *J. natn. Cancer Inst.*, **40**, 231.

PILCH, J. H. & RIGGINS, R. S. (1966) Antibodies to Spontaneous and Methylcholanthrene-induced Tumors in Inbred Mice. *Cancer Res.*, **26**, 871.

RAN, M. & WITZ, I. P. (1970) Tumor-associated Immunoglobulins. The Elution of $IgG_2$ from Mouse Tumours. *Int. J. Cancer*, **6**, 361.

SINCLAIR, N. R. ST. C. & CHAN, P. L. (1971) Relationship between Antibody-mediated Immunosuppression and Tolerance Induction. *Nature, Lond.*, **234**, 104.

SJÖGREN, H. O., HELLSTRÖM, I., BANSAL, S. C. & HELLSTRÖM, K. E. (1971) Suggestive Evidence that the Blocking Antibodies of Tumour-bearing Individuals may be Antigen–Antibody Complexes. *Proc. natn. Acad. Sci. U.S.A.*, **68**, 1372.

SMITH, R. T. (1972) Possibilities and Problems of Immunologic Intervention in Cancer. *New Engl. J. Med.*, **287**, 439.

TACHIBANA, T., WORST, P. & KLEIN, E. (1970) Detection of Cell Surface Antigens on Monolayer Cells. *Immunology*, **19**, 809.

THOMSON, D. M. P. & ALEXANDER, P. (1973) A Cross-reacting Embryonic Antigen—the Membrane of Rat Sarcoma Cells which is Immunogeneic in the Syngeneic Host. *Br. J. Cancer*, **27**, 35.

THOMSON, D. M. P., STEELE, K. & ALEXANDER, P. (1973) The Presence of Tumour-specific Membrane Antigen in the Serum of Rats with Chemically-induced Sarcomata. *Br. J. Cancer*, **27**, 27.